# Assessment on the effectiveness of vessel-approach regulations to protect cetaceans in Australia: A review on behavioral impacts with case study on the threatened Burrunan dolphin (*Tursiops australis*)

**DOI:** 10.1371/journal.pone.0243353

**Published:** 2021-01-19

**Authors:** Helena Puszka, Jeff Shimeta, Kate Robb

**Affiliations:** 1 Marine Mammal Foundation, Hampton East, Victoria, Australia; 2 Centre for Environmental Sustainability & Remediation, RMIT University, Melbourne, Victoria, Australia; University of Missouri Columbia, UNITED STATES

## Abstract

Vessels cause considerable disturbance to cetaceans world-wide, with potential long-term impacts to population viability. Here we present a comprehensive review of vessel impacts to cetacean behavior in Australian waters (2003–2015), finding inadequate protections to be in place. The majority of these studies found trends of decreased animal travel and resting behavioral states as well as low compliance to regulations, and they recommended further regulatory action such as greater enforcement or monitoring, or passive management strategies. As a case study, we conducted the first field assessment of vessel compliance with the *Wildlife (Marine Mammal) Regulations 2009* in Gippsland Lakes, Australia, and provide the first assessment of the endangered Gippsland Lakes Burrunan dolphin (*Tursiops australis*) population’s behavioral ecology. Dolphin behavior and vessel regulation compliance data were collected during boat-based surveys of Gippsland Lakes from July 2017 to January 2018, with a total of 22 dolphin group sightings resulting in 477 five-minute point samples. 77% of dolphin sightings involved vessel interactions (within 400 m), and 56 regulation breaches were observed. These breaches were most severe in summer (mean = 4.54 breaches/hour). Vessels were found to alter dolphin behavior before, during, and after interactions and regulation breaches, including increased mating (mate guarding) and milling behavioral states, and increased ‘fish catch’, ‘high leap’ and ‘tail slap’ behavioral events. These behavioral changes may indicate masking of the dolphins' acoustic communication, disturbance of prey, increased dolphin transition behaviors, and/or induced stress and changes to group structure (including increased mate guarding). While our results provide evidence of short-term altered behavior, the potential for long-term effects on population dynamics for this threatened species is high. In the context of reported inadequate cetacean protection Australia-wide, our management recommendations include greater monitoring and enforcement, and the utilisation of adaptive management.

## Introduction

Although there has been a fundamental shift in attitudes towards cetaceans in Australia, as seen in bipartisan support for cetacean protection, and in Australia playing a key role in the International Whaling Commission (IWC) [1], cetaceans in Australian waters still face substantial threats. Of the 34 cetacean species occurring in Australian waters, six are listed as Threatened in the International Union for Conservation of Nature (IUCN) Red List [2] and 23 are listed as data-deficient, which may constitute a threat in itself [3]. Globally, vessel and noise disturbance constitute key threats to cetaceans [4, 5]. These anthropogenic impacts are often unregulated and appear to be growing in severity [6, 7]. While whale watching has long been presented as benign or beneficial to wildlife [8–10], several papers critique this representation and highlight the potential harm caused by vessels [8, 11, 12].

An animals’ initial response to anthropogenic disturbance is often behavioral, and these responses can impact the distribution, reproduction and survival of a population [13–16]. The population consequences of disturbance (PCoD) framework links short-term changes in individual behavior and physiology to potential long-term effects on population dynamics [17]. The conceptual framework, outlined in New, Clark [17] and Pirotta, Boon [18], explores the effects of exposure to a stressor with physiological and behavioral change, both chronic (individual health; internal factors that affect fitness and homeostasis [17]) and acute (individual vital rates; survival, reproductive success, and growth rate), and how these may affect population dynamics. This framework can readily be applied to investigating vessel-related disturbances to marine mammals.

Vessel disturbance on cetaceans include a wide range of behavioral changes, with potential impacts to core biological activity and physiology [19–23]. Behavioral disturbance may lead to diversion of energy from fitness-enhancing behaviors such as parental care or foraging [14] and can contribute to a population’s vulnerability to recovery or extinction, particularly for small populations [16]. As well as the more direct impacts of incidental catches, vessel strikes and mortality [24–29], vessels cause disturbance through three of the disturbance categories listed by Tuomainen and Candolin [13]: the auditory environment [30, 31]; the visual environment [32, 33]; and changes to habitat size, structure and connectivity [34]. The auditory environment is used by most marine mammal species for communication, foraging and navigation [33, 35, 36], and the frequencies used by these animals can often coincide with those used by vessel engines [37, 38]. In some cases, dolphins have altered both their behaviour and their whistle structure when interacting with tour vessels, possibly allowing more effective communication in a reduced acoustic space and mitigating masking affects [39–41]. Critically assessing and addressing the impacts of vessels to cetaceans should be made a priority in the conservation and management field.

Cetaceans are protected from vessel disturbance by legislation in most, but not all, Australian coastal an offshore waters, with National Guidelines addressing various ways people can legally watch cetaceans [42]. In Commonwealth waters, people must comply with the *EPBC Regulations*, whilst in coastal waters (<3 nautical miles) State and Territory regulations may apply (S1 Table). The National Guidelines state “To protect whales and dolphins and achieve 'best practice’ in whale and dolphin watching, interactions must allow animals to move freely without being chased or harassed if they choose not to interact” and outlines regulations in order to “minimise potential impacts” [42]. While these guidelines and regulations are designed for the protection of these cetacean populations, global studies indicate that marine-mammal approach regulations are frequently breached [43–45]. To date, no comprehensive review of Australian regulations and vessel impact has been undertaken. The aim of this study was to provide an assessment of Australian vessel regulations with regards to cetacean protection. This assessment was conducted through a comprehensive review of vessel approach regulations, cetacean behavioral responses to vessels, and resultant management recommendations in Australia. A case study of Burrunan dolphin (*Tursiops australis*) behavioral response to vessels was conducted to contribute to this assessment.

The Burrunan dolphin is a recently described *Tursiops* species [46] thought to be restricted to southern and south-eastern Australia, with distinct morphologic and genetic divergence, leading to isolated populations across the known range [46–51]. Though the species has not been confirmed by the Marine Mammalogy’s Committee on Taxonomy [52, 53], a larger body of evidence now exists supporting their divergence and uniqueness, further validating the Burrunan as a separate species to the common and Indo-Pacific bottlenose dolphins (*T*. *truncatus* and *T*. *aduncus*, respectively), using mtDNA regions [54], concatenated mtDNA/nuDNA sequences [55], the mitogenome [56–58], and more recently by a time-calibrated molecular phylogeny of Certiodacyla [59]. While the ecology of other bottlenose dolphin species has been studied in depth in Australia for over three decades, little remains known about the Burrunan dolphin.

Only two known resident Burrunan dolphin populations have been identified in the state of Victoria: Port Phillip Bay and the Gippsland Lakes [46–48]. The Gippsland Lakes and Port Phillip Bay populations are estimated at 65 and 120, respectively [46, 48], with the effective population size (those contributing genes to the next generation) reported to be 65.5 individuals in Gippsland Lakes/Tasmania and 81.5 individuals in Port Phillip Bay [48]. Both populations reside in coastal environments adjacent to major human centres, a habitat type considered high risk due to the proximity to anthropogenic impacts [46, 60].

The Burrunan dolphin is yet to be listed, or categorized, under the *EPBC Act* or IUCN Red List due to data deficiencies; however, it is listed as ‘Endangered’ under the State of Victoria’s *Flora and Fauna Guarantee Act 1988* [61] and is vulnerable to extinction due to several different factors relating to exposure to threats, data deficiency, low genetic diversity and low population sizes [48], high mercury levels [60], and increased risk from pathogens and contaminants [62]. Small localised populations may be at high risk of extinction through demographic and genetic stochasticity [63], particularly if they occur close to urban areas where anthropogenic threats abound [64]. Anthropogenic activities, such as cetacean-based tourism or recreational boating, can impact dolphins through physical presence, non-compliance to regulations and acoustic disturbance [65–68]. Such disturbances can negatively affect the long-term viability of small resident populations [69].

**Fig 1:**
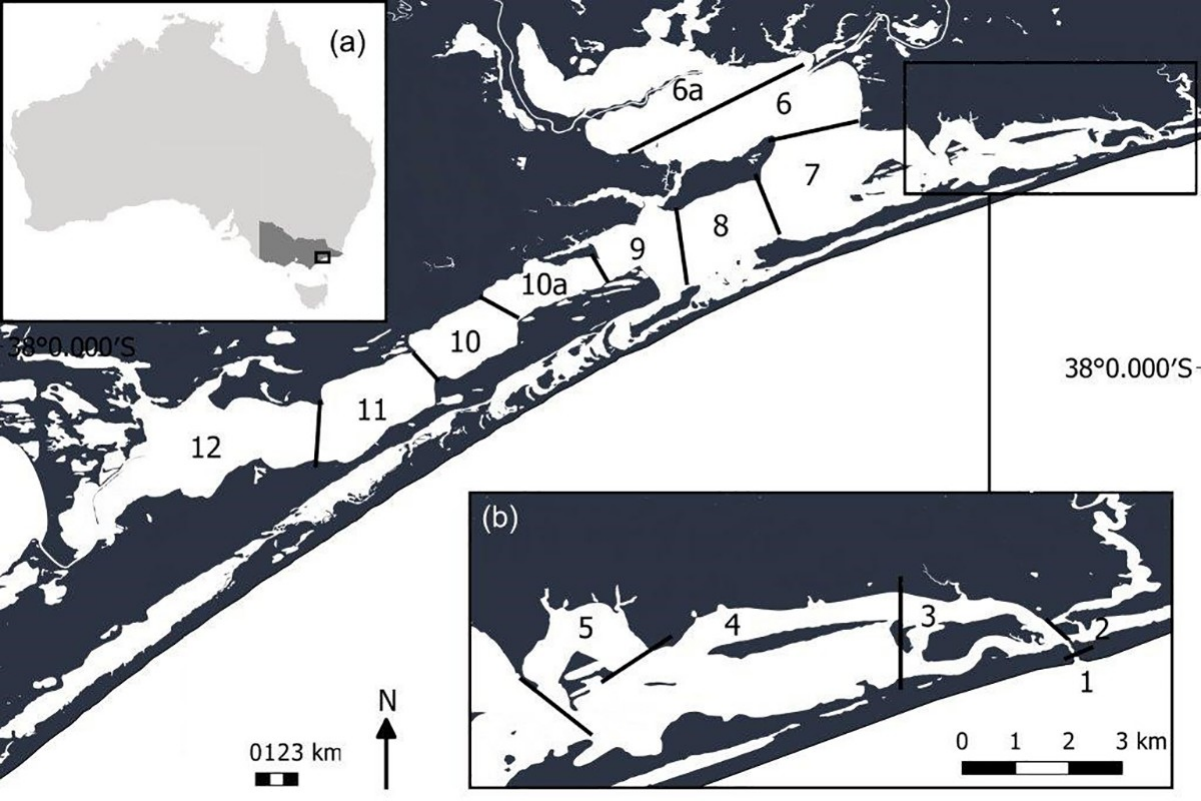
Map of Gippsland Lakes, Australia, showing zones allocated for the study.

Our case study focussed on the population of Burrunan dolphins in Gippsland Lakes, Victoria (37° 49’ to 38° 12’S, 147° 04’ to 148° 08’E, Fig 1), an Australian estuarine system and Ramsar protected site [70]. A variety of vessel types (S2 Table) operate in the lakes system, and the area is acknowledged to be a region exposed to anthropogenic impacts from boating, recreational fishing and tourism [71]. The Burrunan dolphin is listed as high priority value for management and, despite vessel disturbance being listed as a threat [72], there is no quantitative information on vessel impacts to this population, nor on vessel compliance with the *Wildlife (Marine Mammal) Regulations*. It is currently unknown whether vessel interaction and potential behavioral disturbance play a role in the vulnerability of this small, genetically isolated population of endangered animals. This study provides the first assessments of vessel regulation compliance in Gippsland Lakes, and addresses the first step in PCoD to quantify the behavioral responses of an individual to a stressor, in this case, Burrunan dolphin response to vessel interactions (<400m) and violations (<100m).

## Materials and methods

### Literature review

A comprehensive literature review was conducted of all studies of cetacean behavioral responses to vessels in Australian waters published in 2003–2018. Literature was compiled from the online search engines, Web of Science (https://apps.webofknowledge.com) and Google Scholar (https://scholar.google.com), using the key terms "dolphin", "whale", "cetacea", "vessel", "boat", "traffic", "behavior/behaviour", "disturbance", "regulation", "management", and "Australia". Common themes relating to both cetacean behavioral response and management recommendations were identified and documented.

A comprehensive literature review was also conducted on vessel approach regulations. Regulation information and documentation was sourced from the webpages of the Australian Government’s Department of Environment and Energy and relevant Environment Department webpages of all Australian State and Northern Territory governments. The most recent versions of regulations were reviewed for their prescribed prohibited zone and penalty details [70, 73–76].

### Burrunan Dolphin case study

#### Boat-based observation

Boat-based surveys were conducted across the Gippsland Lakes, in daylight hours during Austral Winter (July) and Spring (September) 2017, and Summer (December-January) 2017–2018. Survey periods were chosen to capture seasonal differences and peak (summer) and off-peak tourism periods. Surveys were only conducted on days of low wind (< 15 knots) and good visibility, enabling a 600 metre ‘survey-zone’ [77, 78]. A 2C research vessel, 5.7m Ensign 570 powered by 90 hp Mercury engine, was driven along pre-determined line and zig-zag transects across the Gippsland Lakes system (Fig 1) at depths ≥2 m, at ≥10 m from shore. Continuous horizon scans were conducted to sight Burrunan dolphins, with two to four researchers on board. To minimise potential impacts, the orientation and speed of the animals was observed and then matched by the research vessel; the vessel travelled slowly when possible, and the engine was turned off or placed into idle when possible [79, 80]. The study utilised opportunistic observations of vessel-dolphin interaction rather than controlled experimental vessel approaches. Methodologies were approved by the Victorian State Government’s (Agriculture Victoria) Wildlife and Small Institutions Animal Ethics Committee (WSIAEC 33.14) and RMIT University Animal Ethics Committee, and conducted under Victoria State Government Wildlife Act 1975 Research Permit (Permit number 10008600).

Once dolphins were sighted, observers recorded dolphin group behavioral state (Table 1) and vessel interaction period (Table 2) through point sampling every five minutes. Dolphin behavioral events (Table 1) and vessel violation category (Table 2) were recorded through continuous sampling [81, 82]. Vessel observation included 11 vessel types to provide an assessment of vessel impacts across industries (fishing, tourism, and recreational) (S2 Table). Focal study was conducted on whole dolphin groups rather than individual dolphins to maximise sample size. A group was defined as more than one dolphin within 800 m of each other engaging in the same behavioral state and, if travelling, travelling with the same heading. The primary behavioral state was defined as the behavior in which > 50% of the group was engaged. In the final survey period, vessel behavior was observed continuously to account for the high vessel traffic during this period. The minimum observation period for vessel violation category (before, during or after vessel interaction or violation) was five minutes.

**Table 1:** Behavioral states and key behavioral events of the Burrunan dolphin.

Behavioral state	Definition^1^
Forage	Dolphins involved in any effort to pursue, capture, and/or consume prey, as defined by observations of fish chasing (*herding*), coordinated deep diving and rapid circle swimming. Prey frequently observed at the surface during foraging activity of the dolphins.
Mating	Dolphins observed engaged in body contact with chasing and “belly to belly” behaviors. Penis visible. Herding behavior of separating a female and herding of multiple males. Increased flanking and mate guarding. Increased surface activity, including fast chase.
Mill/Rest^2^	Dolphins exhibited non-directional movement, frequent changes in heading prevent animals from making headway in any specific direction.
Social	Dolphins observed chasing or engaged in any other physical contact with other dolphins (excluding mother–calf pairs). Aerial behaviors such as leaping frequently observed.
Travel	Dolphins engaged in persistent, directional movement, making noticeable headway along a specific compass heading.
Behavioral event	Definition
Fish catch	Either an attempted or successful catch of a fish, fish may be visible in animal’s mouth, thrashing movements at the surface
Flanking	One or more animals pressing up against the side(s) of another, usually herding behavior of a female by males
Leap (normal, high, and/or synchronised)	Airborne forward progress of at least one body length while in the dorsal position (single animal).
	High leap is an acrobatic >1 m leap above water level.
	Synchronous behavior is 2, 3, 4, or ≥5 animals performing the same behavioral event at the same time, within one body length of each other.
Pod split	One or many animals leave the pod, > 1km distance
Tail slap	Flat and noisy contact of the caudal section on the water surface

^1^Terms and definitions were adapted from Shane, Wells [83].

^2^ Mill and Rest were combined due to the difficulty of distinguishing these two states.

**Table 2:** Vessel disturbance categories assigned to each five minute sample.

Interaction	Definition
Before^1^	No vessel interaction observed
During	Any vessel(s) under power, sail or paddle within 400 m of dolphin focal group^2^
Recovery^3^	Any period ≤20 min after vessel interaction in which there is no vessel interaction
After	Any period after Recovery in which there is no vessel interaction
Violation	Definition
Before	No vessel violation observed
During	Vessel(s) violate approach distance or approach angle regulations^4^
Recovery	Any period ≤20 min after vessel violation in which there is no vessel violation
After	Any period after Recovery in which there is no vessel violation

^1^Before-during-after format based on similar studies [80, 84].

^2^ Based on Lusseau [85].

^3^20 min recovery period based on Hawkins [86].

^4^Determined by the *Wildlife (Marine Mammal) Regulations 2009 [87].*

When a pod split occurred (a dolphin group splitting into two or more groups), the decision rule was to alternate between following the larger or the smaller group for each occasion [82]. In total, observations from three observers were used for this study. Behavioral observation was conducted for a minimum of 30 minutes [88, 89] and maximum of 2 hours, and ended either when: (a) observers lost sight of the dolphins; (b) sampling conditions were no longer safe (due to high winds, low sunlight or fatigue); or (c) dolphins exhibited disturbance behaviors towards the research vessel (‘tail slaps’ or increased avoidance).

#### Data and statistical analysis

Data underwent a series of processes to ensure accuracy but maintain optimal sample size (S1 Fig). All behavioral data were collected by one trained observer to minimise observer error, and were validated by the senior observer. Six pod size categories were used: 1–5, 6–10, 11–20, 21–30, 31–50, and 50+ dolphins [90]. Key behavioral events (Table 1) were chosen from the ethogram (Table 2) for analysis. An adapted ‘before-during-after’ analytical design [91] was used in this study. The data were divided into a single-exposure dataset (only including data from the first vessel interaction and first vessel violation observed in the sighting), and a repeated-exposure dataset (including data from the whole sighting). Mean vessel interactions (and violations) per hour of sighting were calculated by dividing the total recorded number of interactions (and violations) from unique vessels by the total dolphin sighting hours. The objective for this case study was to assess if the variation in Burrunan dolphin surface behavior in Gippsland Lakes is driven by both seasonal differences and/or vessel disturbance. To test this, multivariate analyses of variance (MANOVAs) were run in SPSS (IBM SPSS Statistics 25) using the variables listed in Table 3 [92, 93]. The Tukey Honestly Significant Difference test (Tukey HSD) was applied as a post-hoc test to identify significant differences between means.

**Table 3:** Independent and dependent variables used in MANOVA tests.

Independent variables	Categories	Dependent variables	Categories
Season (Austral)	Winter, Spring, Summer	Event frequency, per dolphin	Fish catch, leap (high), leap (synchronised), leap (total), surging, tail slap
Vessel interaction category (repeated exposures)	Before, during, recovery, after	Event frequency, whole group	Change direction, flanking, pod split
Vessel interaction category (single exposure)	Before, during, recovery, after	Predominant state frequency	Forage, mating, mill, social, travel
Vessel violation category (repeated exposures)	Before, during, recovery, after	Secondary state frequency (forage, mating, mill, social, travel)	Forage, mating, mill, social, travel
Vessel violation category (single exposure)	Before, during, recovery, after	Vessel violation frequency (hour) ^1^	n/a (continuous variable)

^1^ Vessel violation frequency was tested with ANOVA (as opposed to a MANOVA), as it was a single dependent variable.

## Results

### Literature review

A review of Australian vessel approach regulations found that cetaceans are legally protected from vessel disturbance in most but not all Australian coastal areas. These regulations are based on the Australian National Guidelines for Whale and Dolphin Watching 2005 (and now 2017) which were developed to ensure consistency across Australian jurisdictions for marine mammal regulations. The guidelines suggest minimum approach distances for cetaceans as well as prescribing details of suggested vessel speed limits and angles of approach (S1 Table). Most Australian regions prescribe a 50 m vessel approach distance for dolphins and a 300 m jet ski approach distance to cetaceans. All regions prescribe a 100 m approach distance for whales. Monetary penalties vary greatly, from zero in Northern Territory and Tasmanian waters and waters 3 nautical miles from the Australian coastline, to up to $132,000 in New South Wales waters.

The search for studies on cetacean behavioral responses to vessel traffic in Australia 2003–2018 found 15 papers, all of which were reviewed with a focus on cetacean behavioral response results (Table 4). All studies suggested some degree of behavioral response to vessel traffic, and the majority of studies (nine) showed changes to multiple behavioral categories. The most commonly found behavioral response was change to travel, found in seven studies (Table 4). Five of these studies specifically found a decrease in travel frequency. Other commonly found responses, found in four studies each, were decreased rest, increased avoidance behavior, and changes to group structure (Table 4). The studies also found decreased social behavior, decreased abundance, increased milling, and changes to surface behavior and habitat use. Notably, Bejder, Samuels [94] found a significant population decline over 4.5 years in the Shark Bay (Western Australia) bottlenose dolphin population due to the presence of merely two dolphin-watching boats. Vessel traffic in Australia produces a variety of behavioral responses in Australian cetacean populations, with a trend of disruption to travel and group cohesion observed.

**Table 4:** Cetacean behavioral responses to vessel traffic from studies of Australian waters 2003–2018.

	Case study		Decreased				Increased		Changes to			
Study	Species	Location	Forage	Rest	Social	Abundance	Avoidance^1^	Mill	Travel^2^	Group structure	Surface behavior	Habitat use
Arcangeli and Crosti [95]	Common bottlenose dolphin (*Tursiops*. *truncatus*)	Bunbury, WA	x	x					x	x		
Bejder, Samuels [96]	Indo-Pacific bottlenose dolphin (*T*. *aduncus*)	Shark Bay, WA							x	x		
Bejder, Samuels [94]	*Tursiops* sp.	““				x						
Cribb and Seuront [97]	*Tursiops* sp.	Port Adelaide River-Barker Estuary, SA										
Filby, Stockin [66]	Burrunan dolphin (*T*. *australis)*	Port Phillip Bay, VIC					x					
Filby, Christiansen [79]	““	““	x					x				
Lemon, Lynch [98]	Indo-Pacific bottlenose dolphin (*T*. *aduncus*)	Jervis Bay, NSW							x		x	
Marley, Salgado Kent [99]	““	Swan-Canning Rivers, WA					x^3^					
Marley, Salgado Kent [100]	““	Fremantle Inner Harbor, WA		x	x				x			
Scarpaci, Nugegoda [101]	*Tursiops* sp.	Port Phillip Bay, VIC	x							x		
Seuront and Cribb [102]	Indo-Pacific bottlenose dolphin (*T*. *aduncus*)	Port Adelaide River-Barker Estuary, SA										
Stamation, Croft [103]	Humpback whale (*Megaptera novaeangliae*)	South coast NSW					x				x	
Steckenreuter, Harcourt [14]	Indo-Pacific bottlenose dolphin (*T*. *aduncus*)	Port Stephens, NSW	x	x					x			
Steckenreuter, Harcourt [69]	““	““										X^4^
Steckenreuter, Moller [104]	““	““	x	x	x		x	x		x		
Total (15 studies)			5	4	2	1	4	2	5	4	2	1

^1^ Includes increased dive duration.

^2^ A variety of changes to travel (eg. erratic travel, changes to travel patterns), not including decreased travel.

^3^ Found at one high-traffic site, but not at another.

^4^ Hypothesized.

Our review of management recommendations with regards to vessel impacts to cetacean behavior in Australia provides a list of scientifically-informed management solutions (Table 5). Five of our 18 reviewed studies, all from Victoria or New South Wales, found low compliance to regulations. Two Victorian and one New South Wales studies stated that existing management practises were passive or inadequate. Almost half of the studies (eight), also exclusively from Victoria and New South Wales, recommended increased enforcement of regulations. The majority of studies [13] from across Australia recommended increased monitoring of cetacean populations and vessel impacts. Five studies from across Australia, including three from Western Australia, recommended the application of adaptive management techniques.

**Table 5:** Management findings and recommendations from studies of cetacean behavioral responses to vessels in Australian water, 2003–2018.

		Findings		Recommendations											
Study	Study location	Low compliance	Existing management inadequate/ passive	Monitoring	Increased enforcement	Adaptive management	Precautionary/ conservative approach	Judicious management (fines)	Exclusion zones	Limits on interaction duration	Regulations more clearly written	Education for:	Tour operators	Tourists	Recreational vessel users
Arcangeli and Crosti [95]	Bunbury, WA			x		x									
Bejder, Samuels [96]	Shark Bay, WA			x		x									
Bejder, Samuels [94]	““			x		x	x								
Blewitt [105]	n/a (Australia-wide review)			x		x									
Cribb and Seuront [97]	Port Adelaide River-Barker Estuary, SA			x											
Filby, Stockin [66]	Port Phillip Bay, VIC	x	x	x	x			x							
Filby, Christiansen [79]	““				x		x	x					x		
Howes, Scarpaci [68]	““	x	x		x			x							
Kessler and Harcourt [106]	Sydney, NSW	x			x										x
Lemon, Lynch [98]	Jervis Bay, NSW			x											
Marley, Salgado Kent [100]	Swan-Canning Rivers, WA			x											
Scarpaci, Dayanthi [107]	Port Phillip Bay, VIC	x			x								x	x	
Scarpaci, Nugegoda [108]	““	x			x						x				
Scarpaci, Nugegoda [101]	““			x	x										
Stamation, Croft [103]	South coast NSW			x			x			x			x	x	
Steckenreuter, Harcourt [14]	Port Stephens, NSW			x											
Steckenreuter, Harcourt [69]	““			x		x			x						
Steckenreuter, Moller [104]	““		x	x	x		x		x						
Total (18 studies)		5	3	13	8	5	4	3	2	1	1		3	2	1

### Burrunan Dolphin case study

From July 2017 to January 2018, a total of 22 dolphin sightings were made, 17 of which involved vessel interaction (Table 6). In total, 477 five-minute behavioral observations were recorded, 411 of which were able to be used in analysis. Every independent variable (including four measures of vessel activity and the variable "season") had statistically significant effects on most or all of the dependent measures of dolphin behavior (events and states) (Table 7). Only three of the 20 null hypothesis were not rejected (Table 7). Thus, vessels and seasonal variation both significantly impacted Burrunan dolphin behavior in Gippsland Lakes. Note that all references to seasons are Austral seasons.

**Table 6:** Survey effort for boat-based surveys. The research vessel has been excluded from vessel counts.

Season (Austral)	Winter	Spring	Summer	Total
Survey days	7	6	8	21
Surveys with dolphin sightings	5	6	5	16
Field effort (h)	44.55	34.97	51.02	131
Total dolphin sightings	9	8	5	22
Dolphin sightings containing no vessel interaction	4	1	0	5
Dolphin sightings containing vessel interaction	5	7	5	17
Mean vessel interactions per hour of sighting	6.64	4.88	30.88	14.40
Mean vessel violations of the *Regulations* per hour of sighting	0.72	0.82	4.54	1.92
5 minute behavioral samples	91	207	179	477

**Table 7:** P values from Wilks’ lambda MANOVA tests.

Independent/Dependent variables	Events (per dolphin)	Events (whole group)	Predominant states	Secondary states
Vessel interaction status (single exposure)	<0.001	0.023	<0.001	0.381
Vessel violation status (single exposure)	0.034	<0.001	<0.001	0.048
Vessel interaction status (repeated exposures)	<0.001	0.047	0.035	0.540
Vessel violation status (repeated exposures)	0.551	0.001	<0.001	0.002
Season	<0.001	0.036	<0.001	<0.001

**Fig 2:**
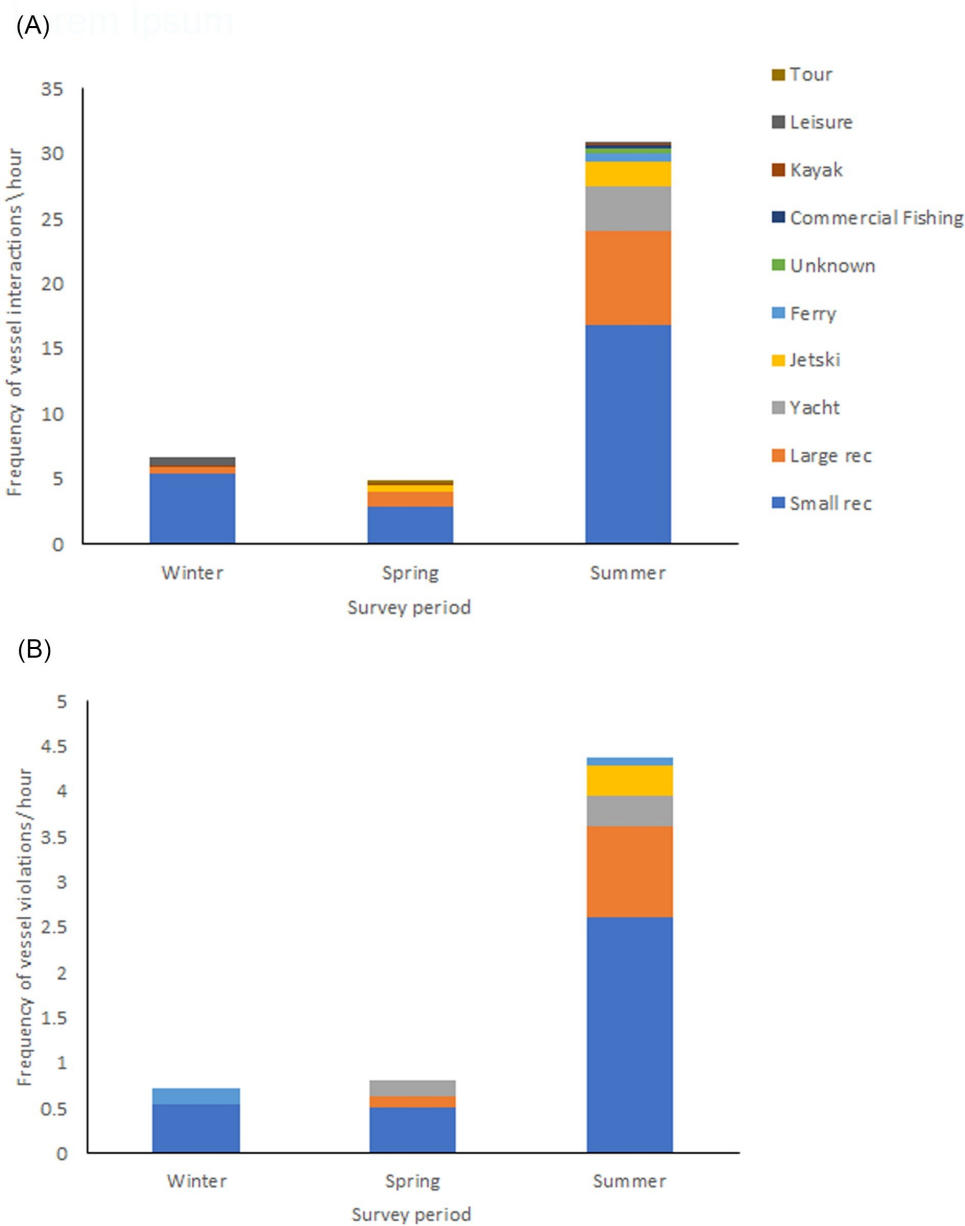
Frequency of (a) vessel interaction, and (b) vessel violations for each season. Rec = recreational vessel (small ≤ 6 m, large > 6 m). See ‘5 minute behavioral samples’ (Table 6) for sample size.

**Fig 3:**
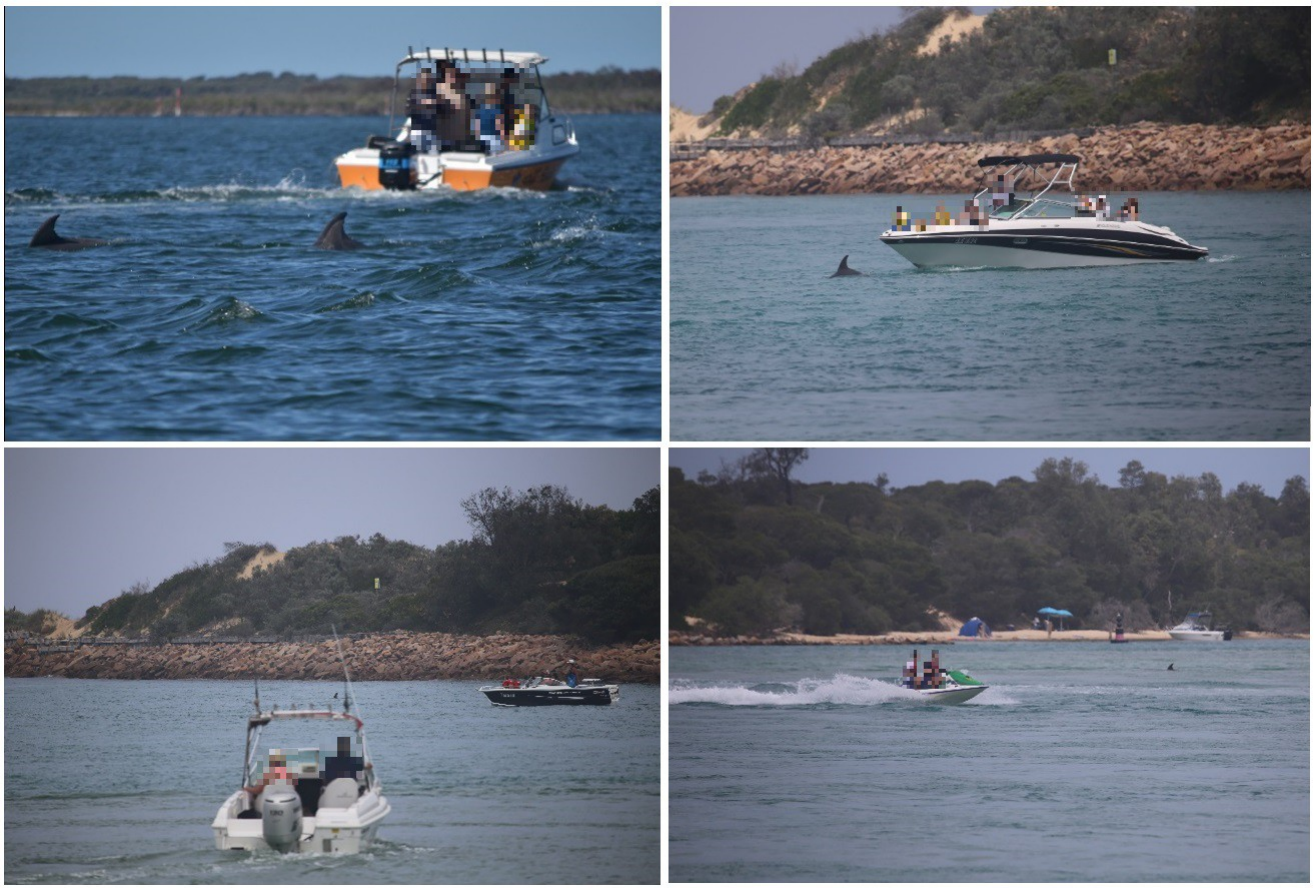
Examples of vessel violations of the *Wildlife (Marine Mammal) Regulations* observed on survey.

All vessel types were observed in Gippsland Lakes across all seasons. Vessel interactions with dolphins occurred across all seasons, and the majority of interactions were undertaken by small recreational vessels, followed by large recreational vessels, yachts and jet skis (Fig 2A). Interaction frequency was significantly higher in summer than winter and spring, with a mean of 30.89 vessel interactions observed per hour of summer dolphin sighting (Fig 2A), Similarly, the majority of vessel violations of Victorian *Regulations* were undertaken by small recreational vessels, followed by large recreational vessels, yachts and jet skis (Fig 2B). Violation frequency was also significantly higher in summer, with a mean of 4.54 vessel violations observed per hour of summer dolphin sighting (Fig 2B). Violations by small reactional vessels included close approaches < 5m (Fig 3).

**Fig 4:**
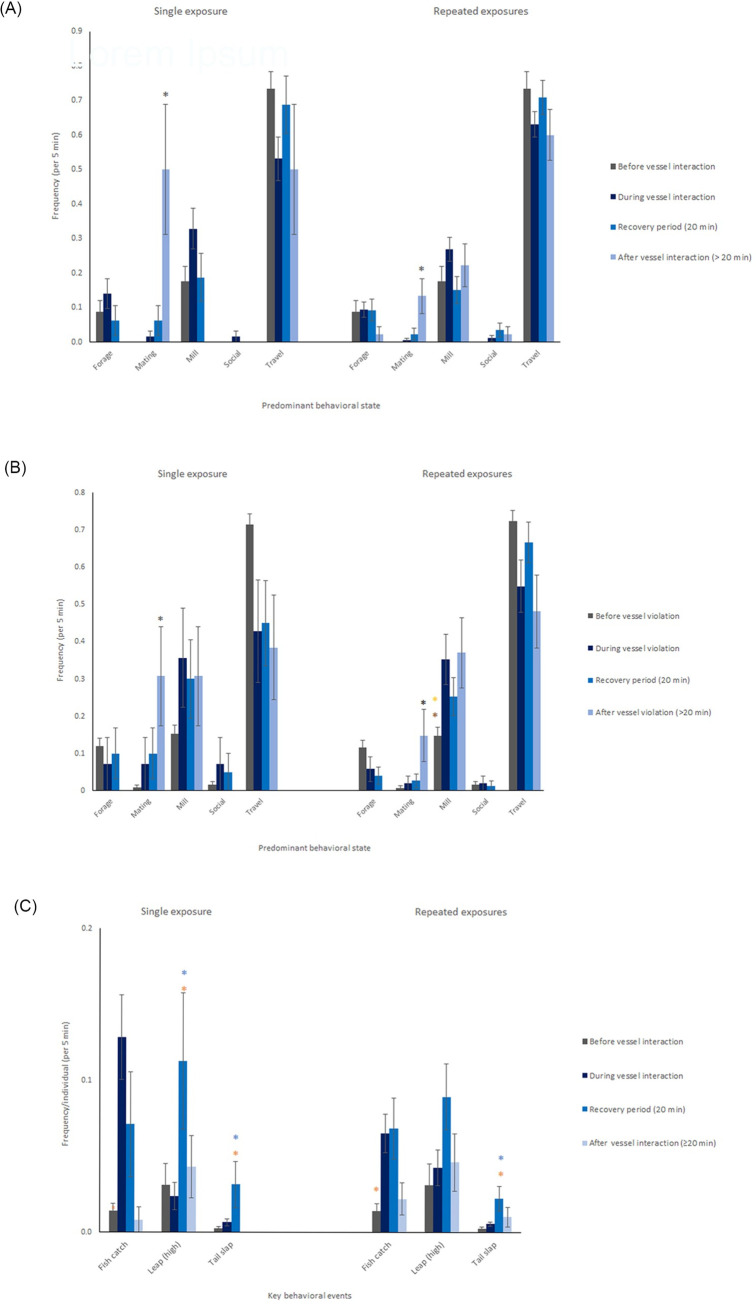
Frequency (mean ± s.e.) of (a) predominant behavioral states for vessel interaction status, (b) predominant behavioral states for vessel violation status, (c) key behavioral events per individual dolphin for vessel interaction status. Within each behavioral state or event, asterisks indicate statistically significant difference (p<0.05, Tukey HSD), and asterisk color denotes the vessel violation categories from which it differs; blue denote before, orange denotes during, brown denotes after. Black asterisks indicate significant difference to all other vessel violation categories. See ‘5 minute behavioral samples’ (Table 6) for sample size.

Behavior of the Burrunan dolphin population showed statistically significant differences for differing levels of vessel disturbance (MANOVAs, p < 0.05, SI4). Mating behavior was seen to increase after both vessel interaction and vessel violation (Fig 4A). Mating behavior increased significantly in the after-vessel-violation period (Fig 4B). Milling behavior increased during and after vessel violation for repeated exposures only (Fig 4B). ‘Fish catches’ significantly increased during vessel interaction (Fig 4C). ‘High leaps’ and ‘tail slaps’ significantly increased during the recovery period of single-exposure vessel interactions only (Fig 4C).

## Discussion

A comprehensive review of both vessel approach regulations, and impacts of vessels on cetacean behavior was undertaken, with on focus on Australian waters (2003–2018). We aimed to assess the current state of regulations, compliance, and adequacies of those regulations. Further we conducted a field assessment of vessel compliance with the *Wildlife (Marine Mammal) Regulations 2009* in Gippsland Lakes, Australia, and provide the first assessment of the endangered Gippsland Lakes Burrunan dolphin (*Tursiops australis*) population’s behavioral assessment in relation to vessel approaches.

### Management recommendations

The broad reach of vessel disturbance to cetacean behavior found in our literature review, including trends of decreased travel and rest, suggests that vessels are a significant hazard to Australia’s cetacean populations and that current regulations and/or enforcement are not adequate. Authors of cetacean behavioral studies in Victoria and New South Wales have deemed vessel regulations to be passive and inadequate [66, 68, 104], and Australia-wide studies have suggested a range of improved management approaches [14, 66, 69, 94–97, 101, 103–107, 109]. The management findings of our literature review are reflected in our case study.

The National Oceanic and Atmospheric Administration’s recent global review of tourism impacts to marine mammals [5] lists the following common management strategies for minimising vessel impacts:

1. Increase enforcement of guidelines and regulations;
2. Revisit viewing distance and vessel speed guidelines;
3. Increase education and awareness;
4. Redesign management systems, and
5. Implement time-area closures or marine protected areas.

Howes, Scarpaci [68] have characterised dolphin conservation in Victoria as reliant on the third listed item, education and awareness, as the principle management strategy. Self-regulation and education do not seem to have successfully protected cetaceans in Port Phillip Bay, Victoria [108, 110]. Some international studies have concluded that education and outreach, named ‘passive actions’, are ineffective for improving vessel compliance with regulations [111, 112], while the first listed item, the presence of police enforcement, proves effective [108, 113]. Constantine [114] names the Western Australian government's decision to withdraw two dolphin-watching permits in Shark Bay as the most effective management strategy, a decision which came after a significant impact from the dolphin watching tours was shown. Severe penalties such as this must be considered if other options prove ineffective. Future education and outreach strategies must coincide with a simplification and clarification of the written regulations, as recommended by Scarpaci, Nugegoda [108].

The following changes to vessel management regulations in Australia are recommended:

1. Further monitoring of vessel impacts to cetaceans, including behavior and population viability assessments;
2. Implementation of an adaptive management approach;
3. Review of vessel approach distance regulations according to research findings; and
4. Increased enforcement of regulations.

Adequately protecting cetaceans from vessel impacts provides several challenges, including those of data deficiency for many Australian species, and complex socio-economic influences and stakeholder interests [115]. Further monitoring, the most common recommendation from our management recommendations review (Table 5), will ideally address this data deficiency. It is necessary for further monitoring to link to an adaptive management approach, in which continual scientific research on unique habitats, populations and/or disturbances informs unique fit-to-order management approaches [116]. Bejder, Samuels [96] advocate adaptive management, as it “eschews the one-final-solution strategy, and instead enables managers to move forward in the face of uncertainty, multiple variables, and/or incomplete information about cause-and-effect relationships”. As an extreme example of action taken in adaptive management, Steckenreuter, Harcourt [69] describe incorporating a Total Exclusion Zone for all vessel traffic, when indicated necessary by monitoring data. Since our case study was undertaken, there has been a review of the *Wildlife (Marine Mammal) Regulations* in Victoria, with new *Regulations* published in 2019 [117]. It is noted that an adaptive management approach has been incorporated, enabling greater responsiveness and flexibility to identify, add or amend areas important to marine mammals, which previously required ‘slow and administratively burdensome’ regulatory amendment [118].

Vessel approach distance regulations must be based on research findings such as those from our Gippsland Lakes case study. Behavioral analysis needs to be ongoing, and in conjunction with population viability assessments, to provide a comprehensive assessment of long-term vessel impacts and seasonal variation. An adaptive management approach is relevant to the situation in Australia, and it is recommended in circumstances which involve both uncertainty and controllability [96, 119].

### Burrunan Dolphin case study

Variation in Burrunan dolphin surface behavior in Gippsland Lakes was shown to be driven by both seasonal differences and vessel disturbance. This overall result is consistent with previous studies and indicates that vessel disturbance constitutes a considerable threat to the population.

**Fig 5:**
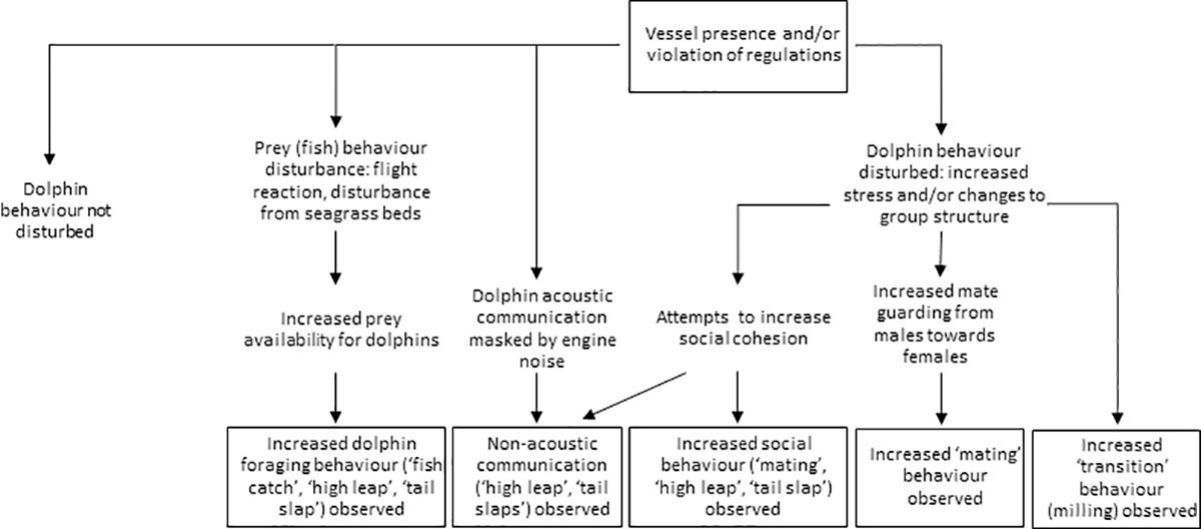
Potential cause-and-effect relationship for observed dolphin behavioral responses to vessel disturbance. Text in a box indicates observations, text without a box indicates untested hypotheses for mechanisms of vessel disturbance.

Changes to foraging, mating and social behaviors are all common animal reactions to anthropogenic disturbance [13]. Surprisingly, this study showed increases in foraging, mating and social behaviors. There are several mechanisms potentially underlying this specific set of behavioral responses. Meanings of behavioral events can be ambiguous, as events can have multiple functions [120, 121]. The behavioral responses observed may be due to vessels interrupting all behavioral activity, causing stress and changes to group structure (including increased mate guarding), masking dolphin acoustics through engine noise, and/or the disturbance of fish (Fig 5). Whilst increased mate guarding after vessel disturbance can be noted as a potential ‘negative’ change in behavior, an increase in ‘fish catches’ may be seen as an advantageous response to vessel disturbance. Multiple mechanisms of disturbance may be at play and have the potential to interact with one another.

The observed increase in milling behavior (Fig 4) is consistent with Filby, Christiansen [79] and Peters, Parra [84] analyses of Burrunan dolphin behavioral response to anthropogenic disturbance (vessels and swimmers, respectively). The increase in milling behavior may constitute a minor short-term ‘pause’ in dolphin behavior, or a more severe impact to this population if vessel disturbance is frequent or severe enough to prevent dolphins from engaging in fitness-related activities [13].

Mating behavior (Table 1) was shown to significantly increase after vessel interaction and violation (Fig 4). This contrasts with previous global studies which found anthropogenic activities leading to decreases in *Tursiops* species mating behavior [122, 123]. Many studies place mating behavior as a sub-category of social behavior [124–126]. It is important to note that in this study, mating behaviour has been documented not only by the act of mating but also increased mate guarding, flanking and increased surface activity. Mate guarding is a mating strategy in which males spatially sequester females, often to prevent rivals from mating with the female [127]. In coalitionary mate guarding, stable alliances of two to three male bottlenose dolphins may aggressively herd an individual female to establish and maintain consortship [128]. In this case study, male dolphins may have increased mate guarding to assert control over individual females during vessel disturbance events when the dolphin group is undergoing transition behavior or experiencing stress and/or changes in group structure. Interestingly, increased mating behavior was found in the Port Phillip Bay Burrunan dolphin population as a response to vessel disturbance [79], and it is possible that a mate guarding response was being observed in these instances as well. Increased mate guarding behavior may be beneficial for population reproductive rates, and/or it may place harmful strain on females, as in Wallen, Patterson [129] where mate guarding was found to impact the energy budgets and behavioral and spatial ecology of female bottlenose dolphins.

The increased social and mating behaviors observed may also indicate group attempts at increasing social cohesion after group structure is disturbed. Mating behavior has been seen to increase upon the fusion of dolphin groups [130, 131], and Neumann [130] has suggested mating could indicate an affiliative social ‘greeting ritual’ for dolphins. Increased social behavior was also seen in increased ‘high leaps’ and ‘tail slaps’ (Fig 4C). Filby, Christiansen [79] also found increased socialising from the Port Phillip Bay Burrunan dolphin population in response to swim-with-dolphin tours. Previous studies have found changes to bottlenose dolphin group structure [80], decreased group cohesion [132], and increased group cohesion [133] as a response to vessel disturbance. The increased mating and social behavior could also be linked to a stress response. Social behavior can indicate stress, as close contact with a group enhances vigilance and predator detection [84, 134, 135].

Multiple studies have found that vessel engine noise can mask marine mammal acoustic communication [136–138], and it is likely that vessels operating within 5 m of Burrunan dolphins (Fig 3) at least partially interrupted Burrunan dolphin acoustics. ‘Leaps’ and ‘tail slaps’ have been considered as forms of percussive communication used to communicate in noisy environments [93]. The observed increase in ‘high leaps’ and ‘tail slaps’ in the recovery period of vessel interactions (Fig 4C) may be explained as an alternative form of group communication. This theorised use of non-vocal communication is likely to constitute a maladaptive approach as the energetic impact of ‘high leaps’ and ‘tail slaps’ is likely greater than that of vocal signalling.

Increased ‘fish catches’, ‘high leaps’ and ‘tail slaps’ (Fig 4C) may be a result of increased prey disturbance due to vessel presence. Key fish prey for the Gippsland Lakes Burrunan dolphin are known to inhabit seagrass beds in Gippsland Lakes [71, 139]. Previous studies show altered behavior of fish caused by noise pollution (such as boat engines), including changes to swimming activity [140], increased flight reactions and stress response [141, 142], unstructured fish schools, decreased anti-predator defence, and greater vulnerability to predation [141]. It is likely that vessel presence caused disturbance of fish schools, leading to increased prey availability for the dolphin population, and may explain the increased ‘fish catch’ behavior observed during vessel interaction (Fig 4C). The increase in ‘fish catch’ could be argued to constitute a successful adaptive response to disturbance. There is a risk, however, that behavioral adaptations mask emerging ecological issues (defined as ecological issues that are in development or underway [16]); for instance, Burrunan dolphins could face increased risk of boat strikes due to close proximity to vessels during these prey disturbance events. The increased ‘high leaps’ and ‘tail slaps’ may also be explained by this mechanism of prey disturbance, rather than (or as well as) a form of non-vocal communication, as these events are also known to be used by dolphins as prey herding behavior [143, 144]. Increased ‘tail slaps’ with vessel interaction could also be stress-related [145] and a sign of irritation, annoyance or aggression [138, 146]. ‘Tail slaps’ have been found to be a dolphin behavioural response to vessel approaches [86, 147, 148] and sound [138].

#### Repeated exposures

The behavioral response results show some consistency and some differences between single and repeated vessel exposures (Fig 4). Analysis of these differences and similarities may illuminate potential cumulative behavioral responses [30, 149], habituation of this population to vessel disturbance [150, 151], or potential data analysis limitations. Most statistically significant results were consistent between the two datasets, as seen in increased mating after vessel interaction and violation, increased ‘fish catches’ during vessel interaction, and increased ‘tail slaps’ in the vessel interaction recovery period (Fig 4).This consistency in single and repeated exposure results, as well as the strong overall behavioral response results, may indicate that this population is not habituated to vessel impact at this level of disturbance [30]. Milling frequency was lowest before vessel violation for both single and repeated exposure results; however, the difference was only statistically significant for repeated exposures (Fig 4B). This difference may indicate a cumulative milling response due to continued behavioral disturbance to a dolphin group throughout the day. Alternatively, these differences in single and repeated exposure results may indicate the need for further testing and greater sample size for a more comprehensive analysis of the behavioral responses of this population.

#### Consequences of vessel disturbance

In order to assess population-level consequences of disturbance, following PCoD, the duration and intensity of exposure, and the proportion of the population that is exposed to the stressor, is required [18]. In this study we have identified clear and significant changes in behavior to vessels; however, further investigation understanding the above aspects is required to fully understand the population-level and long-term consequences, making particular note that closed populations have been shown to be more sensitive to disturbance [17, 18, 152].

Interpretation of an altered behavior due to a disturbance event is often difficult because the behavior can have positive, neutral, and/or negative aspects. For example, increased mating can lead to increased energetic cost to males and increased aggression towards reproductive females, but it may also lead to an increase in the physical act of mating and may increase reproductive output. This risk in interpretation, or misinterpretation, can not only have consequences to management and mitigation but to the long-term viability of a population. The Burrunan dolphin behavioral responses found in this study may constitute adaptive or maladaptive responses to vessel disturbance, dependent on whether they lead to a reduction in fitness [13]. Regardless, the potential impacts of violations of the *Wildlife (Marine Mammal) Regulations* to the Burrunan dolphin population was found to be severe, particularly from small recreational vessels in the summer tourism period (Fig 2). Actions of vessels included extended follows of dolphin pods at close range (< 5 m) and directly driving over dolphin groups, and in summer the dolphin groups often faced repeated regulation violations in a short duration of time. Whilst there have been no documented ship or vessel strikes in the Gippsland Lakes, the level of non-compliance to the *Regulations* pose an increase threat of strikes. In addition, vessels that did not violate the *Regulations* but travelled within 400 m of dolphins were also found to alter dolphin behavior. This indicates that the minimum approach distance of 100 m does not adequately protect this population.

Christiansen and Lusseau [153] argue that cetaceans may be able to appropriately adapt to occasional vessel presence, but repeated vessel disturbance provides the animals with little opportunity to adapt. The Gippsland Lakes Burrunan dolphin population is exposed to repeated vessel disturbance year-round. When the potential impacts to fitness as well as the known effects of noise pollution on cetaceans are considered, this vessel action constitutes a severe risk to the already threatened dolphin population.

Behavioral responses to vessels may expend energy that could be used for other core biological activities [154], and as previously discussed, changes to core biological activity and physiology can contribute to a population’s vulnerability to recovery or extinction, particularly for small populations [16]. The behavioral responses observed may be due to vessels interrupting all behavioral activity, causing stress and changes to group structure (including increased mate guarding), masking dolphin acoustics through engine noise, or the disturbance of fish (Fig 5). These mechanisms may have taken place simultaneously, and have the potential to interact and lead to cumulative disturbance effects to this population.

#### Gippsland lakes management recommendations

The Burrunan dolphin is listed as a Natural Value to the Gippsland Lakes [155] and, despite vessel disturbance being listed as a threat to the Gippsland Lakes Burrunan dolphins [54], prior to this study there was no quantitative information on vessel impacts to this population, nor on vessel compliance to the *Regulations*. This study has found mean vessel interactions and violations were high, with as much as 30.88 mean vessel interactions and 4.54 violations per hour of sighting (Table 6). As such, Victorian regulations regarding vessel approaches, in order to “reduce the risk of disturbance to natural behaviours”, appear to be inadequately recognised by vessel users and are inadequately enforced. Global studies indicate that marine mammal approach regulations are frequently breached [43–45], and in a study of swim-with-dolphin tours in the Ticonderoga Bay Sanctuary Zone (Port Phillip Bay), Howes, Scarpaci [68] found 100% of observed tours (n = 104) breached minimum approach distances. Regulations are enforced through patrols by wildlife officers, *ad hoc* monitoring by the Department of Environment, Land, Water and Planning and Parks Victoria, and reporting through the public [87]. It is duly noted that enforcement operations and legal processing of non-compliant cases are resource-dependent and can be cost-prohibitive. The consequences of non-compliance and management inaction are high, and in this instance applying the precautionary principle (defined as “when an activity raises threats of harm to human health or the environment, precautionary measures should be taken even if some cause and effect relationships are not fully established scientifically” [156]) is appropriate, rather than waiting for harmful long-term effects to become clear [157, 158].

In Victoria, marine mammal tour permits exist for both sighting-seeing and whale (dolphin) swim tours, whilst the Limited Areas Permits (Port Phillip Bay) are currently under review. Specific to the Gippsland Lakes, dolphin-watching tourism is acknowledged to be important to the economy by Department of Sustainability and Environment [87], with the industry noted as ‘emerging’ [71], and without implementation of management strategies in Gippsland Lakes, vessel disturbance is likely to intensify. There are currently no permitted sightseeing or swim permits issued for operators for the Gippsland Lakes, however there are numerous operators advertising dolphin sighting seeing as a key attraction feature. These operators, according to the Regulations, should adhere to the 100m approach distance.

The loss of only a few Burrunan dolphins could have catastrophic impacts on the population [45]. Disturbance to Burrunan dolphins in Gippsland Lakes should be considered a likely considerable impact following the Australian Government’s Matters of National Significance impact guidelines [159]. Regarding critically endangered or endangered species, it is deemed that an action is likely to have a significant impact if there is a real chance or possibility that the action will lead to a long-term reduction in population, reduce area of occupancy and disrupt the breeding cycle. It is also recommended that the Burrunan dolphin be included in the *EPBC Act* List of Threatened Fauna [73] and Biologically Important Areas in Gippsland Lakes are recognised in the Australian Government’s National Conservation Values Atlas.

## Conclusion

Our case study demonstrated that vessel regulations have not adequately protected the Burrunan dolphin in Gippsland Lakes, while our literature review demonstrated that cetaceans are not adequately protected across Australia. Multiple management strategies need to be employed to ensure vessel compliance with regulations, including increased enforcement, monitoring, and adaptive management. This case study also contributes to the limited knowledge of the endangered Burrunan dolphin species, and provides crucial information on an anthropogenic threat to the Gippsland Lakes population. Violations of the *Wildlife (Marine Mammal) Regulations* were found to be severe in the summer months, and both vessel interaction and violation were found to cause a significant change in behavior. There is a high risk that altered behavior, in response to vessel disturbance, constitutes a maladaptive response. For an already threatened population, energetic impacts may have long-term consequences for population survival.

## Supporting information

S1 Fig Treatment of data from raw data to data analysis. ‘N’ refers to number of five minute samples in each dataset. Solid-line boxes indicate datasets created, dashed-line boxes indicate procedures undertaken.
(DOCX)

S1 Table Australian regulations concerning vessel approach distances to cetaceans. (DOCX)

S2 Table Vessel definitions for vessels found in Gippsland Lakes. (DOCX)
