# Supplementary material for: Assessment on the effectiveness of vessel-approach regulations to protect cetaceans in Australia: A review on behavioral impacts with case study on the threatened Burrunan dolphin (Tursiops australis)
Source: PLoS One. 2021 Jan 19;16(1):e0243353. doi: 10.1371/journal.pone.0243353 (PMC7815133; doi:10.1371/journal.pone.0243353)
Supplement: S1 Fig — ‘N’ refers to number of five minute samples in each dataset. Solid-line boxes indicate datasets created, dashed-line boxes indicate procedures undertaken. (DOCX) [file pone.0243353.s001.docx]

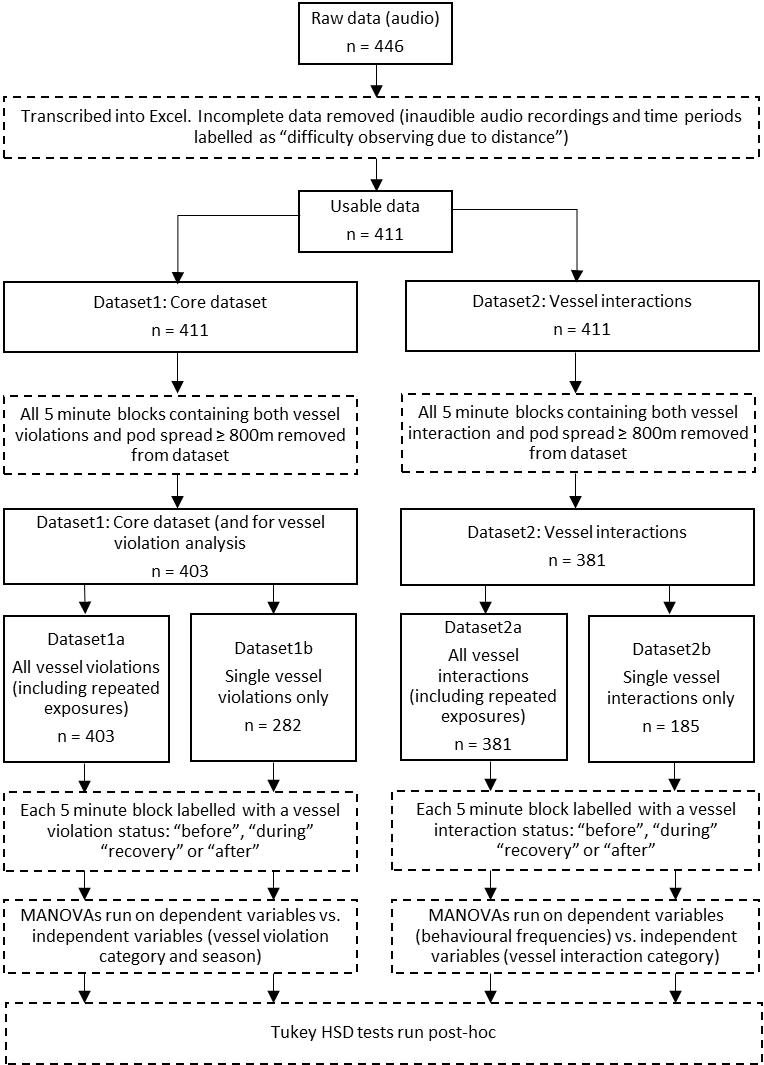


Figure S1. Treatment of data from raw data to data analysis. ‘N’ refers to number of five minute samples in each dataset. Solid-line boxes indicate datasets created, dashed-line boxes indicate procedures undertaken
