# Supplementary material for: Assessment on the effectiveness of vessel-approach regulations to protect cetaceans in Australia: A review on behavioral impacts with case study on the threatened Burrunan dolphin (Tursiops australis)
Source: PLoS One. 2021 Jan 19;16(1):e0243353. doi: 10.1371/journal.pone.0243353 (PMC7815133; doi:10.1371/journal.pone.0243353)
Supplement: S1 Table — (DOCX) [file pone.0243353.s002.docx]

Table S1. Australian regulations concerning vessel approach distances to cetaceans.

|  |  | Prohibited zone (m) | | |  |
| --- | --- | --- | --- | --- | --- |
| Region | Regulation name | Whales | Dolphins | For prohibited vessels (eg. jet skis) | Penalty^1^ |
| 3 nautical miles from Australian coastline | *Australian National Guidelines for Whale and Dolphin Watching 2017 & Commonwealth Environment Protection and Biodiversity Conservation Act 1999* | 100 | 50 | 300 | None |
| New South Whales | *Biodiversity Conservation Regulation 2017* | 100 | 50 | 300 | Tier 2 monetary penalty^2^ up to $132,000 |
| Northern Territory | None (refers to national guidelines) | 100 | 50 | 300 | None |
| Queensland | *Nature Conservation (Wildlife Management) Regulation 2006* | 100 | 50 | 300 | Maximum 165 penalty units ($21,540.75) |
| South Australia | *National Parks and Wildlife (Protected Animals - Marine Mammals) Regulations 2010* | 100 | 100 | 300 | Up to $100,000 |
| Tasmania | None (refers to national guidelines) | 100 | 50 | 300 | None |
| Victoria | *Wildlife (Marine Mammal) Regulations 2009* | 100 | 100 | 300 | 20 penalty units ($3,223.80) |
| Western Australia | *Biodiversity Conservation Regulations 2018* | 100 | 100 | 100 | $5,000 |

^1^ Brackets indicate calculated fine in AUD at time of writing.

^2^ For approach distances or interfering with marine mammals (including harassing, chasing, and herding).
