# Supplementary material for: Assessment on the effectiveness of vessel-approach regulations to protect cetaceans in Australia: A review on behavioral impacts with case study on the threatened Burrunan dolphin (Tursiops australis)
Source: PLoS One. 2021 Jan 19;16(1):e0243353. doi: 10.1371/journal.pone.0243353 (PMC7815133; doi:10.1371/journal.pone.0243353)
Supplement: S2 Table — (DOCX) [file pone.0243353.s003.docx]

Table S2. Vessel definitions for vessels found in Gippsland Lakes.

| Vessel type | Description^1^ |
| --- | --- |
| Commercial fishing | Large commercial fishing vessel with an inboard motor. Large rear deck space and an enclosed front cabin |
| Dredger | Backhoe dredger mounted on a spud-stabilised pontoon. Uses a hydraulic excavator to dredge material, but is not self-propelled |
| Hire | Small recreational vessel emblazoned with names of known local boat hire companies. These boats may be driven by people with no boat license |
| Jet ski | Personal water craft which is jet-propelled |
| Kayak | Light, canoe-like boat with a watertight frame in which a paddler sits with legs forward, using one paddle with two blades |
| Large recreational | Fibreglass-hulled boat with either inboard or large outboard motor. Length 6m or more. Equipped with either partially or fully enclosed cabin |
| Small recreational | Small, fibreglass or aluminium hulled boat with an outboard motor. Under 6m in length. Open top with forward controls or a centre console; designed to stay on the water for short periods of time (i.e. day trips) |
| SUP | Stand-up paddleboard. Propelled by paddling by rider using one paddle with one blade |
| Tour | Displacement hull with an inboard motor. Equipped with extensive deck space, fully-enclosed cabin, and entertainment facilities for tourism |
| Water police | Large vessel clearly marked as Water Police |
| Yacht (sailboat) | Small yacht with both sail and inboard engine capabilities |

^1^ Descriptions based on Marley *et al.* (2017).
